# Eikenella Corrodens, Cause of a Vulvar Abscess in a Diabetic Adult

**DOI:** 10.1155/2007/63565

**Published:** 2006-12-20

**Authors:** Nefise Öztoprak, Ülkü Bayar, Güven Çelebi, Mustafa Basaran, Füsun Cömert

**Affiliations:** ^1^Department of Infectious Diseases and Clinical Microbiology, Faculty of Medicine, Zonguldak Karaelmas University, 67600 Zonguldak, Turkey; ^2^Department of Obstetrics and Gynecology, Faculty of Medicine, Zonguldak Karaelmas University, 67600 Zonguldak, Turkey; ^3^Department of Microbiology and Clinical Microbiology, Faculty of Medicine, Zonguldak Karaelmas University, 67600 Zonguldak, Turkey

## Abstract

We report a case of *Eikenella corrodens* causing vulvar abscess in a diabetic patient. *Eikenella corrodens* is a slow growing, nonmotile, facultative anaerobic, Gram-negative bacillus which is commensal of the oral cavity, intestinal and genital tracts. The most common clinical sources of this organism are human bite wounds, head and neck infections and respiratory tract infections. In our knowledge, the presented case is the first report of *Eikenella corrodens* causing vulvar abscess in a diabetic patient.

## 1. INTRODUCTION

*Eikenella corrodens* is a slow growing, nonmotile, facultative anaerobic, Gram-negative bacillus which is commensal of the oral cavity, intestinal and genital tracts [1–3]. The most common clinical sources of this organism are human bite wounds, head and neck infections and respiratory tract infections [1, 2, 4]. Eikenella species have also been shown to cause endocarditis, intraabdominal infection, pancreatic abscess, arthritis, vertebral osteomyelitis, discitis, orbital cellulites, thyroid abscess, brain abscess, liver abscess and genitourinary infections [1–3, 5–8]. According to our research, this is the first report of *Eikenella corrodens* causing vulvar abscess in a diabetic patient in English literature.

## 2. CASE

A diabetic 55 year old woman was admitted to Zonguldak Karaelmas University emergency clinic with a complain of an insidious onset of swelling in the left part of vulva, a pain radiating to the left inguinal region, chills and high fever over a week. She has had hypertension and type II diabetes mellitus and has used oral anti-diabetic and anti-hypertension drugs for 8 years. She had no history of previous trauma, operation, underlying skin lesion, use of foreign body or human bite on her vulvar region. Two days after onset, her left part of the vulva became swelling and the lesions color turned to red. Even though she had used amoxicilline-clavulanic acid and ornidasole for three days, the lesion on the vulva and pain worsened.

**Figure 1:**
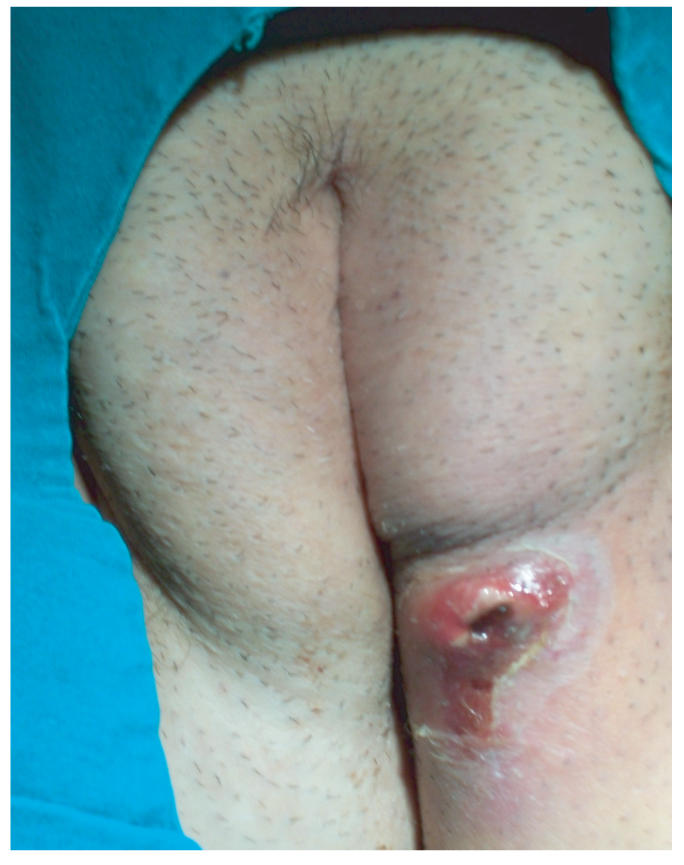
Vulvar abscess (The lesion is 5 × 4 cm in size, red, and swollen).

In clinical examination her fever 38°C, pulse 96/dk, blood pressure 160/80 mmHg, in the left part of the vulva there was an erode lesion in the certain part of the inflammatory halo. The lesion was 5 × 4 cm in size, red, hot, swollen, and painful and fluctuant. Local tenderness and edema were also marked (Figure 1). The borders of the lesion was not elevated and sharply demarcated.

Laboratory values were as follows: WBC count, 18200/ mm^3^ (82% neutrophils, 8% lymphocyte, 8% monocytes) [4000–10000/mm^3^]; erythrocyte sedimentation rate (ESR), 122 mm/hour [0–20 mm/hour]; C-reactive protein, (++++)[negatif]; and blood glucose, 235 mg/dL [70–110 mg/dL].

Vulvar abscess was diagnosed and it was drained. In Gram stain of the pus, granulocytes (80% neutrophil), Gram-negative rods and Gram-positive cocci were detected. Ampicilline-sulbactam 4 × 1 gr IV/day, ciprofloxacin 2 × 400 mg IV/day and local care with 2% eau borique fluid were started empirically. Her oral anti-diabetic drugs stopped and IV insulin treatment started. The blood glucose was in regular limits after IV insulin treatment began. Following drainage, her fever, leucocyt count became normal and, edema, pain and erythema of the lesion subsided.

Eikenella corrodens and metisilline sensitive *Staphylococcus epidermidis* (MSSE) were isolated from culture of the pus. After 3 days of the treatment her fever, white blood cell count was normal, ESR was 100 mm/hour. The patient completed 14 days of treatment with resolution of signs and symptoms. The ESR was 72 mm/hour in the first week of the treatment, 30 mm/hour at the end of the treatment. An informed consent was obtained from the patient to use of her photographs.

## 3. DISCUSSION

*Eikenella corrodens* is commonly found in oral, gastrointestinal, and genitourinary flora. A break in barriers, such as mucous membranes or the skin, can lead to hematogenous spread and serious *Eikenella* infections [2]. It is described that previous or concomitant illness had a greater association with *Eikenella* infections than did a previously healthy status [2]. Diabetes mellitus is one of these underlying conditions; our case is also type II diabetic patient.

*Eikenella* is typically susceptible to many antibiotics, including penicillin G, ceftriaxone, amoxicillin-clavulonic acid, and fluoroquinolones [2]. Some of the *β*-laktamases produced by *Eikenella* are inhibited by clavulonate and sulbactam, however *β*-laktamase production is uncommon at present [1]. Surgical drainage may be more important than antibiotics alone in the management of *Eikenella* infections [2]. Our patient was started on amoxicillin-clavulonate and ornidasole before admitting to our hospital and not responded well until drainage of the abscess. Depending on the location of infection, treatment choice is a combination of surgical management and antibiotics. Our patient has been successfully treated with ampicilline-sulbactam and ciprofloxacin for 14 days.

There are a few literatures reporting that ESR is a valuable test in genital abscess [9, 10]. However, we could not find any study showing correlation between ESR level and vulvar abscess. On the other hand, in a study reported by Paul and Patel including 54 children and adolescents with *E. corrodens* infections elevated ESR was detected for most of the patients and the authors considered that ESR was a better indicator than WBC count for *E. corrodens* infections [2]. In this case, ESR was 122 mm/hour at the onset of the illness and ESR level reduced to 30 mm/hour at the end of the treatment.

*E. corrodens* causes various clinical manifestations especially in diabetic patients and ESR may be a good indicator of Eikenella *corrodens* infections.
